# Air–liquid interface cultures trigger a metabolic shift in intestinal epithelial cells (IPEC-1)

**DOI:** 10.1007/s00418-023-02180-x

**Published:** 2023-02-15

**Authors:** Martin Stollmeier, Stefan Kahlert, Werner Zuschratter, Michael Oster, Klaus Wimmers, Berend Isermann, Hermann-Josef Rothkötter, Constanze Nossol

**Affiliations:** 1 Institute of Anatomy Medical Faculty, Otto-Von-Guericke University, 39120 Magdeburg, Germany; 2 Leibniz Institute for Neurobiology, Otto-Von-Guericke University, 39120 Magdeburg, Germany; 3 Research Institute for Farm Animal Biology, 18196 Dummerstorf, Germany; 4 Institute of Laboratory Medicine, Clinical Chemistry and Molecular Diagnostics, University of Leipzig, 04103 Leipzig, Germany

**Keywords:** Air–liquid interface, ALI, IPEC, Lactate, Glucose, ATP, Metabolism, HIF-1α, Microarray

## Abstract

An improved oxygen availability in air–liquid interface (ALI) cultures of enterocytes of the small intestine seems to be primarily responsible for morphological, metabolic, and functional changes. Intestinal porcine epithelial cells 1 (IPEC-1) are less investigated and are rarely used as model for intestinal barrier but showed a profound change of cell shape during ALI cultivation. We aim to answer the following question: Are the observed morphological effects accompanied by changes in metabolic function? A microarray analysis of submerged culture (SMC) and ALI cultures identified 830 significantly regulated genes. Subsequent functional clustering revealed alterations in 31 pathways, with the highest number of regulated genes in metabolic pathways, carbon metabolism, glycolysis, and hypoxia-inducible factor (*HIF*) signaling. Furthermore, *HIF-1α* as a mediator of a metabolic switch between glycolysis and oxidative phosphorylation showed a trend of increased mRNA levels in ALI in contrast to a reduced nuclear HIF-1α content in the nucleus. Candidate genes of oxidative phosphorylation such as a mitochondrial marker exhibited enhanced mRNA levels, which was confirmed by western blot analysis. Cytochrome C oxidase (*COX*) subunit 5B protein was decreased in ALI, although mRNA level was increased. The oxidation of ferrocytochrome C to ferricytochrome C was used for detection of cytochrome C oxidase activity of isolated mitochondria and resulted in a trend of higher activity in ALI. Furthermore, quantification of glucose and lactate concentrations in cell culture medium revealed significantly reduced glucose levels and decreased lactate production in ALI. To evaluate energy metabolism, we measured cellular adenosine triphosphate (ATP) aggregation in homogenized cell suspensions showing similar levels. However, application of the uncoupling agent FCCP reduced ATP levels in ALI but not in SMC. In contrast, blocking with 2-desoxy-d-glucose (2DG) significantly reduced ATP content in ALI and SMC. These results indicate a metabolic shift in IPEC-1 cultured under ALI conditions enhancing oxidative phosphorylation and suppressing glycolysis.

**Supplementary Information:**

The online version contains supplementary material available at 10.1007/s00418-023-02180-x.

## Introduction

The intestinal tract is characterized by villi and crypts. The growth of both structures increases rapidly postpartum, where growth rate and development depend on luminal nutrient composition (Zabielski et al. 2008). The enterocytes form a highly dynamic barrier between the external (luminal/mucosal) and internal (serosal/vascular) compartment due to (1) formation of highly prismatic enterocytes as a monolayer, (2) microvilli on apical side, (3) expression of tight junction complexes, (4) desmosomes and zonula adherens, and (5) localization on a porous support (basement membrane).

For analysis of the intestinal barrier, continuously growing cell lines allow long-term cultivation but should be characterized by the above-mentioned features. These features are largely dependent on the choice of cell line, growth support, and cultivation technique (Klasvogt et al. 2017; Nossol et al. 2011). The function, differentiation, and morphology of enterocytes are also affected by their metabolism (Brandes and Rezende 2021), and this in turn is influenced by nutrient and oxygen supply. Changes in the cellular microenvironment due to a lack or an improved availability of oxygen/glucose or inflammatory stimuli resulted in a profound change of the metabolic state of the cells. Modifying cultivation techniques via air–liquid interface (ALI) culture improves morphological characteristics in gastric epithelial cells (Ootani et al. 2000) and in enterocytes (Nossol et al. 2011) and is a standard protocol for airway epithelial cells (Cozens et al. 2018) but also changes the metabolic state of the cells (Klasvogt et al. 2017; Kondo et al. 1997).

Intestinal porcine epithelial cells 1 (origin: jejunum and ileum; IPEC-1) and intestinal porcine epithelial cells (origin: jejunum; IPEC-J2) are both originally isolated from newborn piglets and are non-transformed and non-tumour derived (Steube et al. 2012). Especially IPEC-J2 represents a well-established model for the simulation of the intestinal barrier (Geens and Niewold 2011; Guilloteau et al. 2010), while IPEC-1 is little used for studies. Nossol and colleagues (Nossol et al. 2011) showed in their study morphological differences in the cultivation of both cell lines via ALI culture. An ALI cultivation of IPEC-1 resulted in a profound change of cell shape, villin expression, and DNA synthesis. In contrast, IPEC-J2 cells only slightly changed their shape but showed a higher number of microvilli and increased villin expression independent of cultivation technique (Nossol et al. 2011). Furthermore, Klasvogt and colleagues (Klasvogt et al. 2017) analyzed the impact of ALI on IPEC-J2 with a focus on metabolism. Although both cell lines originate from the small intestine, they differ from each other. The aim of the study was to examine the effect of ALI on IPEC-1 and shed light on possible metabolic differences between both cell lines.

## Materials and methods

### Cell culture

Intestinal porcine epithelial cells (a friendly gift from Mariana Roseli, Italy; IPEC-1; ACC 705) (Steube et al. 2012) IPEC-1 were regularly tested and found to be free of mycoplasma contamination (Venor GeM Mycoplasma Detection Kit; Minerva Biolabs, Berlin Germany). Cells were grown as described by Nossol et al. (Nossol et al. 2011) with a seeding density of 1.0 × 10^5^/cm^2^ within the upper compartment of the transwell system (ThinCerts; culture size: 113.1 mm^2^; pore size: 1 µm; polyester, Greiner Bio-One, Kremsmünster, Austria). Cultivation was performed in DMEM F12 (1:1) medium supplemented with 5% fetal bovine serum (FBS), 16 mM 4-(2-hydroxyethyl)-1-piperazineethansulfonic acid (HEPES), 1% insulin–transferrin–selenium (ITS) (all from PAN-Biotech, Aidenbach, Germany), and 5 ng/mL epidermal growth factor (EGF; Biochrome, Berlin, Germany). IPEC-1 were grown at 39 °C in an incubation atmosphere of 5% CO_2_ and 95% relative humidity. At the beginning of the experiment, SMC and ALI culture medium was applied to apical and basolateral compartment. After 10 days of cultivation, FBS supplement to the culture medium was reduced stepwise every second day (2.5%, 1%, 0%). Cells were cultured for 21 more days in FBS-free cell culture medium. SMC cultures were incubated with a liquid column of medium (1 mL/12 wells) and ALI cultures with a thin liquid film on the apical side. Cell culture medium was changed every 2 days. For quantification of glucose and lactate cell culture medium was left on cells for 5 days before retrieving samples.

### Transepithelial electric resistance (TEER)

The integrity of the confluence was checked due to visual inspection of the monolayer and a quantification of transepithelial electrical resistance (TEER) using Millicell ERS (Millipore, Darmstadt, Germany). For TEER measurement, prewarmed medium was added to the apical compartment of ALI for the duration of measurement only (maximum duration 5 min). Between the measurements, the electrode was disinfected using 70% ethanol, washed in AMPUWA, and calibrated in prewarmed FBS-free culture medium. TEER was assessed once a week. We defined a threshold value of 0.5 kΩcm^2^ for all cultured cells to be included in our experiments.

### RNA isolation

IPEC-1 were cultured on 24-well ThinCerts with 10 mm diameter. After withdrawal of the apical and basolateral medium, cells were covered with TRIzol reagent (Invitrogen, Waltham, MA, USA) as described by the manufacturer’s protocol and scraped off the membrane. In the next step, chloroform was added to the cell lysate and supernatant was extracted. RNA was precipitated using isopropanol alcohol. RNA was purified using 75% ethanol and stored in RNA-free water peqGOLD (Peglab, Erlangen, Germany) at −80 °C until further processing.

### Microarray analysis

For microarray analysis, the isolated RNA of at least three independent experiments was further purified employing RNeasy Kit (Qiagen, Germany) and quantity was determined using NanoDrop ND-1000 spectrophotometer (Peqlab, Germany).

The array analysis was performed as described by Diesing et al. (Diesing et al. 2012). Briefly, each GeneChip Porcine Genome Array (Affymetrix, UK) was loaded with 500 ng of purified RNA and analyzed using Affymetrix GCOS 1.3 software according to the manufacturer’s directions. The raw data have been deposited in a minimum information about a microarray experiment (MIAME)-compliant database (Edgar et al. 2002), the National Center for Biotechnology Information Gene Expression Omnibus (http://www.ncbi.nlm.nih.gov/geo), via the accession number GSE202088. Data were inspected for quality before further preliminary processing using the R package “arrayQualityMetrics” (Kauffmann et al. 2009). Data have been normalized via the GC-RMA approach (R package gcrma). Differentially expressed genes were identified and statistically evaluated using R statistical language, and statistical significance was examined by applying a *t*-test; adjusted *p*-values were used for further analyses (*p* < 0.05). Differences in mRNA abundance between the experimental groups were expressed as fold change (FC) and were calculated from least-squares means. The cutoff criteria considered FC > 1.5 and FC < −1.5. For functional analysis of significantly up- and downregulated genes, cellular and metabolic pathways were mapped using DAVID bioinformatics resources.

## Quantitative real-time PCR

Five experiments were carried out for quantitative real-time PCR. The Quibit RNA Assay Kit (Life Technologies, Waltham, MA, USA) was used to measure RNA content. Using the SensiFast TM SYBR/No-ROX One Step Kit (2 ng RNA, 10 pmol of each primer, Bioline, Germany), qualitative real-time PCR was performed according to the manufacturer’s protocol. For further details of utilized primers, see Table 1.

**Table 1:** Used primer pairs

Name	Function	Sequence 5′–3′	Sequence 3′–5′	Temperature (°C)	Efficiency (%)
*β-Actin*	Cytoskeletal protein	tgcactttattgaactggtctca	gtatgaagttcaacgccctgt	61.0	115
*COX-5B*	Cytochrom-c-oxidase subunit 5B	tgatgaggagcaggcgac	gtcggagtccatggttcctt	56.6	109
*18S*	Ribosomal subunit 18S	gcaattattccccatgaacg	aacctaccaaatcactccgg	60.5	98
*GAPDH*	Glyceraldehyde-3-phosphate-dehydrogenase	acccagaagactgtggatgg	ttccagtagggactcgagtt	56.5	128
*GLUT1*	Glucose transporter 1	gagccctgcctagacacttg	agaagatggggttctccacc	62.5	100
*HIF-1α*	Hypoxia-inducible factor	cagctatttgcgtgtgagga	aactttcggaacctaccaaa	62.0	98
*HK2*	Hexokinase 2	ttgaacagcagaccgtcta	ctttcttgcgactcttac	60.0	108
*Mito-marker*	60 kDa component of mitochondria	gcaaactcttccaccaccag	accgacttctactacacctca	62.5	110
*MCT1*	Monocarboxylate-transporter 1	tccatctgttggctgtcat	cgaactaacgtcgaaggaag	58.5	98
*VIM*	Vimentin	tgtcaagatggctctcgaca	ggacttggactccctttggt	58.5	104

For reverse transcription, samples were heated up to 45 °C for 10 min followed by the activation of polymerase (95 °C, 2 min) and 40 cycles of denaturation (95 °C, 5 s, qTower, Analytik Jena AG, Germany) and annealing with elongation (varying temperature according to the primer, 20 s) in alternating order. *β-Actin* and *18S* served as housekeeping genes, and the geometric mean of both was calculated for further analysis. Experiments were carried out in sets of three technical replicates (*N* = 5), whose results were averaged and further mathematically processed using the ΔΔ−CT method.

### Western blot

The medium was aspirated, and the cells were washed with PBS. After adding SDS loading buffer (1 M Tris base pH 6.8, vol. 10% glycerol, vol. 2% SDS, vol. 0.005% bromophenol blue, vol. 5% β-mercaptoethanol), cell lysate was heated up to 95 °C for 5 min for protein denaturation. The Qubit Protein Assay Kit using the Qubit 2.0 Fluorometer (both Invitrogen, Waltham, MA, USA) was used for quantification of protein content.

Forty micrograms of protein sample as well as PageRuler prestained protein ladder (SM0671, Fermentas Wltham, MA, USA) were loaded on an SDS polyacrylamide gel. After electrophoresis, samples were transferred to 0.45 µm PVDF membrane by semidry electroblotting using Trans-Blot SD Semi Dry Transfer Cell (Bio-Rad, Munich, Germany). The BM Chemiluminescence Western Blotting Kit (1:10,000, mouse/rabbit) was employed for protein detection following the manufacturer’s instructions, but milk (5%, RT, 1 h) was used as blocking reagent. For specific protein identification, different antibodies were utilized: mouse-anti-GAPDH 1:2000 (Cell Signalling, Cambridge, UK), rabbit anti-MCT1 1:1000 (Bioss Antibodies, Woburn, MA, USA), rabbit anti-COX5B 1:1000 (Abgent, Danvers, MA, USA), mouse anti-mitochondria 1:500 (abcam, Cambridge, UK), mouse-anti-HIF-1α 1:500 (Novus Biologicals, Abingdon, UK), rabbit-anti vimentin (1:1000, Origene, USA), and mouse anti-β-actin 1:40,000 (Sigma-Aldrich, Munich, Germany). All primary antibodies were incubated overnight at 4 °C and secondary antibody for 2 h at room temperature.

Furthermore, the NE-PER Nuclear and Cytoplasmic Extraction Reagents (Thermo Scientific, Waltham, MA, USA) were utilized to extract proteins from both compartments of the cells. The extraction was executed following the manufacturer’s instructions. IPEC-1 cells were aspirated and incubated with trypsin/EDTA (10 min) to detach the cells of the membrane. In the next step, cells were resuspended in 500 µL FBS-free cell culture medium. Three ThinCerts were pooled and centrifuged (500*g*, 5 min). The cell pellet was resuspended in 1 mL PBS containing protease inhibitor (Roche, Basel, Switzerland), and cell number was determined. In the next step, cell pellet was dissolved in CER I (−20 °C, volume in accordance with cell count), vortexed (15 s), and incubated on ice for 15 min. CER II (−20 °C, volume in accordance with cell count). The application of CER II resulted in the liberation and solution of cytoplasmic proteins, but nuclear integrity was maintained. The supernatant was removed and stored at −80 °C. The pellet was solved in NER I solution, leading to the liberation of the nuclear fraction, which was stored in analogous manner. For the detection of HIF-1α, protein content was determined and 30 µg was compounded with the equimolar amount of SDS loading buffer. Electrophoretic separation was performed on an 8% SDS polyacrylamide gel. As an internal control we used a HeLa cell lysate for HIF-1α western blots, which is commercially available (Novus Biologicals, Germany; data not shown). These cells were incubated with CoCl_2_ for 4 h to induce hypoxia in the cells.

The raw intensities of the protein bands were used for statistical analyses. All experiments were carried out three times. The loading control (β-actin) was utilized for normalization, and the band with the highest raw intensity was used to normalize all other β-actin bands (normalization factor). Subsequently, the normalization factors were applied to the corresponding raw intensities of the investigated proteins. The normalized SMC (control) values were used as reference in the boxplot diagrams.

### Lactate and glucose measurement

IPEC-1 cells were grown on ThinCerts of 15 mm diameter. The medium was altered within the final 5 days of cultivation. No medium was added on the apical side of the ALI culture. Therefore, no lactate or glucose was measured in the apical compartment of the cells. The cell culture medium was fully withdrawn from the cells and transferred into separate tubes according to the compartment. All samples were stored on ice until measurement. Cell-free culture medium was used as blank. Glucose and lactate concentrations were measured on the Cobas C 501 system (Roche). Glucose consumption and lactate production were calculated due to the differences between blank (cell-free cell culture medium) and sample.

### ATP measurement

Cells were cultured on 24-well ThinCerts. One day before ending the experiment, cells were treated with or without carbonylcyanid-4-trifluormethoxyphenylhydrazon (FCCP, 5 µM in DMSO, Sigma Aldrich, Hamburg, Germany) and 2-desoxy-d-glucose (2DG, 5 mM in glucose-free medium) for 24 h. On the day of the experiment, medium was withdrawn and membranes were liberated from the framework. Boiling 4 mM EDTA/100 mM Tris buffer was used to cover the cells and scrape them off the membrane. In the next step, lysate was incubated at 100 °C for 2 min, followed by a centrifugation step (1000*g*, 60 s). Fifty microliters of the supernatant were transferred as triplets onto white 96-well microplates (Greiner Bio-One, Germany) and were stored on ice until further processing. ATP quantification was carried out using ATP Bioluminescence Assay Kit CLS II (Roche, Germany) according to the manufacturer’s protocol (Tecan M200, Tecan, Germany).

### Quantification of cytochrome C oxidase activity

The absorption rate of cytochrome C changed with the state of oxidation. We reduced cytochrome C with 1 M dithiothreitol (DTT). The cytochrome C oxidase of the sample will oxidize reduced cytochrome C. Isolated mitochondria were used in the experiment for measuring cytochrome C oxidase activity. Mitochondria were isolated following the procedure reported by Klasvogt et al. 2017 (Klasvogt et al. 2017). Ten microliters of sample was mixed with 5 μL of cytochrome C DTT solution [2.7 mg cytochrome C from equine heart, 5 μL 1 M DTT (both Sigma, St. Louis, MO, USA) in 1 mL distilled water] and 95 μL of 10 mM Tris–HCl with 120 mM KCl. Absorption was measured at 550 nm for a duration of 60 s at an interval of 10 s. In addition, the absorption of a blank (10 μL 10 mM Tris–HCl with 250 mM sucrose, 3.5 μL cytochrome C DTT solution, 95 μL 10 mM Tris–HCl with 120 mM KCl) was assessed. Enzyme activity was calculated as shown by Eq. (1):

1 $${\mathbf{cytochrome}} \, {\mathbf{C}} \, {\mathbf{oxidase}} \, {\mathbf{activity}} \, \left( {{\mathbf{U}}/{\mathbf{mL}}} \right)\,\, = \,\,\frac{{\left( {{\text{delta absorbance }}\left( {{\text{sample}}} \right) \, - {\text{ delta absorbance }}\left( {{\text{blank}}} \right)} \right){\text{ x }}0.{\text{11 mL}}}}{{0.0{\text{1 mL x 21}}.{84}}}$$

### Immunofluorescence staining

Cells were fixed with ethanol (30 min, 4 °C) and acetone (90 s, −20 °C precooled). Phosphate buffer (PB) 0.1 M was used as wash buffer, and every step was performed three times for 5 min (RT). After washing the cells, they were permeabilized with 0.3% Triton/0.1 M PB for 30 min (RT), blocked with 1% normal goat serum in 0.1 M PB supplemented with 10% bovine serum albumin (30 min, RT), and washed. Primary antibodies were incubated at room temperature (mouse anti-HIF-1α, Novus Biologicals, USA, mouse anti-vimentin, Origene, USA; rabbit anti-tricellulin, Life Technology; all 1:100 in 0.1 M PB) for 2 h. In addition, cells were washed and supplemented with secondary antibody (goat anti-mouse IgG2b Alexa 555, Life Technology, USA; goat anti-mouse Alexa IgG 555, Life Technology, USA; goat anti-rabbit Alexa 488, Invitrogen, USA; all 1:200 in 0.1 M PB/10% BSA) for 1 h at room temperature. After a washing step, cells were stained with DAPI (1:10 in 0.1 M PB) and washed again. Cells were covered with VECTASHIELD and coverslips.

Epifluorescence images were acquired with an Axiovert 200 M inverted microscope equipped with an EC Plan-Neofluar 40×/0.75 objective and an AxioCam MRm digital camera (1388 × 1040, 12 bit). Blue (DAPI), green (Alexa 488), and red (Alexa 594) fluorescence was detected with filter sets FS01 (356/12//395//397; 65 ms), FS44 (475/40//500//530/50; 1.15 s), and FS63 HE (572/25//590//629/62; 210 ms), respectively. All devices were obtained from Zeiss, Jena, Germany.

### Confocal microscopy

Confocal imaging was performed using a Leica TCS SP5 (Leica Microsystems, Wetzlar, Germany). Briefly, the system comprises an upright microscope [DM 6000 CFS with a tandem scanning system (SP5), acousto-optical tunable filters (AOTF) and an acousto-optical beam splitter (AOBS)] equipped with a diode 405 nm laser, an argon laser (laser lines: 458, 476 488, 496, 514 nm), a DPSS 561 laser, and HeNe 594 nm and 633 nm lasers.

The diameter of the confocal pinhole was set to 95.6 µm (Airy 1), and images were taken by scanning the focused laser beam with a galvo mirror through an HCX PL APO CS 63× NA 1.4 oil objective (Leica Microsystems, Wetzlar, Germany) at zoom factor 10 across the specimen. Unidirectional scans were recorded at scan speeds of 700 Hz with line averaging of 6. Confocal image stacks consisting of 97–145 focal planes with an axial step size of 0.13 µm (total depths between 12.1 and 18.1 µm) were digitized with 8 bit depth at 1024 × 1024 pixel resolution. This resulted in *x*, *y*, *z* voxel sizes of 24 nm × 24 nm × 126 nm. Fluorescence signals of the different dyes were detected sequentially (between laser lines) by photomultiplier tubes within two spectral regions [571–639 nm for Alexa 555 (channel 1) and 417–496 nm for DAPI (channel 2)]. With this sequential excitation setting, any crosstalk between channels could be excluded. To address the question of a nuclear localization, individual focal planes or image stacks were checked in *x*–*z* or *y*–*z* direction for the colocalization of immune markers.

After scanning, images were processed for contrast and brightness levels of individual channels using either ImageJ (National Institutes of Health, USA) or Photoshop CS 5 (Adobe. System Inc., San Jose, USA).

### Statistical analysis

Data were checked for normal distribution (Shapiro–Wilk test) and homogeneity of variance (Levene test). In the case of normal distribution, one-way ANOVA (Dunnett’s post hoc test or Dunnett’s T3) or *t*-test was performed, and if data were not normally distributed, a Mann–Whitney *U*-test was done (*p ≤ 0.05; *p ≤ 0.01;**p ≤ 0.001). In the case of multiple testing, an alpha correction was performed.

## Results

### Microarray analysis

**Fig. 1:**
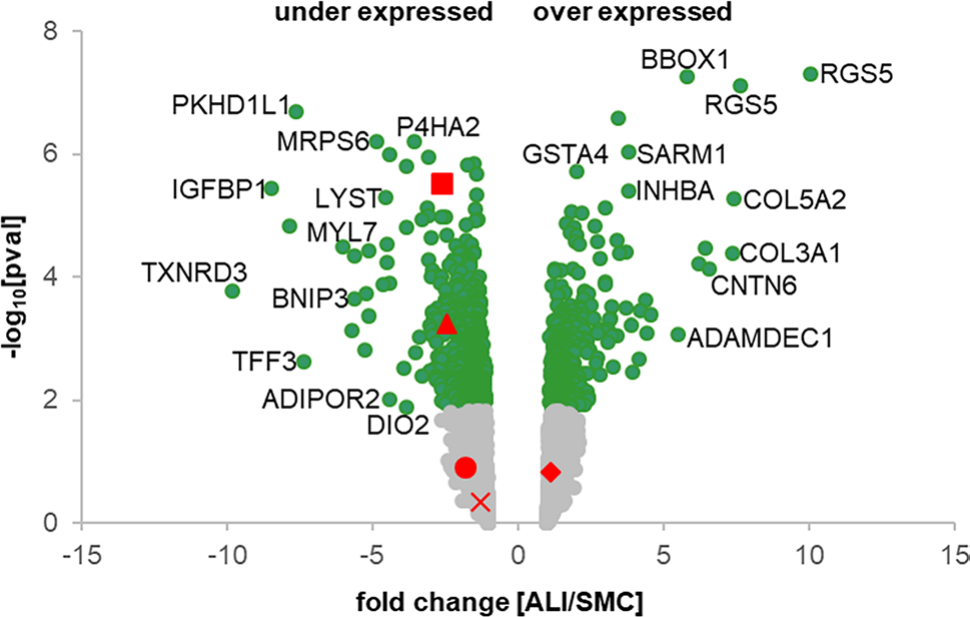
Volcano plot. Differentially regulated genes are represented as gray dots, and significantly regulated genes are marked as green dots. Significantly overexpressed genes are represented on the right side and significantly underexpressed genes are represented on the left side in the diagram. The names of highly under- and overexpressed genes can be seen at the left and right side of the volcano plot. Furthermore, genes of interest are marked in the volcano plot (rhombus: *COX5B*; circle: *HIF-1α*; quadrangle: *VIM*; triangle: *GAPDH*; cross: *HK2*)

Differences in the gene expression of ALI-cultured cells and SMC cells should also be investigated. Therefore, a microarray analysis was performed and ALI-cultured cells were compared with SMC cells. In the first step, differentially regulated genes were presented as a volcano plot (Fig. 1). Significantly regulated genes were labeled as green dots. Genes with a high positive fold change were *RGS5*, *BBOX1*, *COL5A2*, *COL3A1*, and *CNTN6*. Genes that were strongly negatively regulated were *PKHD1L1*, *IGFBP1*, *TXNRD3*, and *TFF3*.

Subsequently, a functional analysis of significantly up- and downregulated genes was performed and cellular and metabolic pathways were mapped using DAVID bioinformatics resources. Our microarray analysis resulted in 909 significantly upregulated and 1396 downregulated genes in the air–liquid interface cultures compared with submerged cultures (*p* ≤ 0.05). For functional analysis, we used genes that were at least 1.5-fold regulated. A total of 300 genes were up- and 530 genes were downregulated. Our functional clustering resulted in 31 regulated pathways. In Table 2, the top ten significantly regulated pathways are shown (Table 2). In first place is HIF signaling, second glycolysis/gluconeogenesis, third metabolic pathways, fourth carbon metabolism, and fifth biosynthesis of amino acids. All genes regulated in the individual pathways can be found in the supplementary material Table S1.

**Table 2:** The search of significantly regulated genes was specified, and genes with 1.5-fold change value were used for further analyses (FC ≥ 1.5; FC ≤ 1.5). In total, 830 genes were significantly regulated (300 up- and 530 downregulated). These genes were used in DAVID to find significantly regulated KEGG pathways in ALI cultures compared with SMC. Overall, 31 pathways were differently controlled. In the table, the top ten regulated pathways are shown with corresponding *p*-values and gene counts

		KEGG pathway	Gene count	*p* value
1		HIF signaling	17	1.5 × 10^–7^
2		Glycolysis/gluconeogenesis	13	2.6 × 10^–7^
3		Metabolic pathways	80	4.9 × 10^–7^
4		Carbon metabolism	14	3.1 × 10^–5^
5		Biosynthesis of amino acids	10	3.0 × 10^–5^
6		Steroid biosynthesis	5	2.7 × 10^–3^
7		Cholesterol metabolism	7	3.8 × 10^–3^
8		Pyruvate metabolism	6	9.1 × 10^–3^
9		Cell cycle	10	1.3 × 10^–2^
10		Drug metabolism—other enzymes	7	1.4 × 10^–2^

### Analysis of genes of interest via qPCR and western blot

**Fig. 2:**
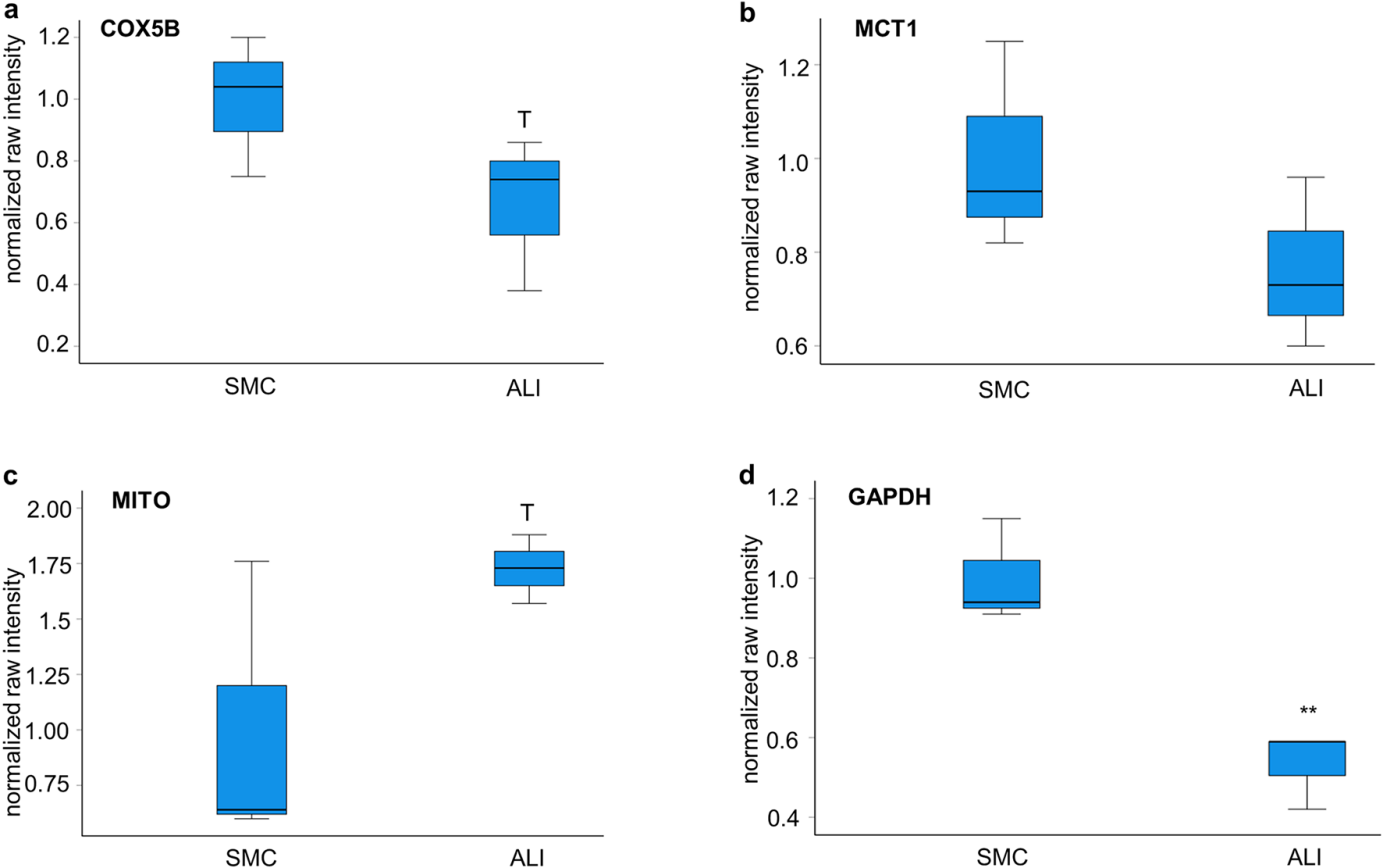
Western blot analyses. We analyzed the raw intensities of our western blots and used the loading control β-actin for normalization (*N* = 3). **a** We detected a trend (*p* = 0.08) of lower COX-5B protein expression in ALI- compared with SMC-cultured cells. **b** The analysis of MCT1 resulted in a lower content in ALI cultures. **c** Subsequently, we analyzed the MITO protein and detected a trend (*p* = 0.096) of increased content in ALI-cultured cells. **d** Furthermore, a significantly decreased GAPDH protein expression (*p* = 0.019) was observed in ALI culture compared with SMC cultures

In the next step, important genes of these pathways, including *HK2*, *GAPDH*, *COX-5B*, *GLUT1*, *MCT1*, *HIF-1α*, and *VIM* (Table 3), were analyzed via qPCR. *COX-5B* showed a trend (*p* = 0.085) of upregulation in ALI on qPCR but a trend of a decreased COX-5B protein expression on western blot analyses of ALI cultures compared with SMC cultures (*p* = 0.08; Fig. 2a). Significantly upregulated genes on qPCR (Table. 3) in ALI compared with SMC were mitochondrial marker (*p* = 0.001) and MCT1 (*p* = 0.022). MCT1 protein was slightly increased in the comparison of SMC with ALI (Fig. 2b). MITO protein also showed a trend of increased protein content in ALIs compared with SMC (*p* = 0.096; Fig. 2c). Glyceraldehyde-3-phosphate dehydrogenase (*GAPDH*) catalyzes the phosphorylation of glyceraldehyde-3-phosphate during glycolysis and was found to be downregulated in ALI (*p* = 0.002). Our western blot analyses (Fig. 2d) confirmed the result of the qPCR. Here, we detected a significantly lower content of GAPDH in ALI compared with SMC cultures (Fig. 2d). *GLUT1* encodes for a transmembrane glucose transporter, which plays a key role in regulation of intracellular glucose. The analysis of *GLUT1* and* HK2 *resulted in no significant regulation in qPCR. All original western blots can be found in the supplementary material as Fig. S1.

**Table 3:** Gene regulation of ALI compared with SMC (100%) (< 100%, downregulated; > 100%, upregulated). Different genes of the metabolic pathways were analyzed via qPCR. *18S* and *β-actin* were used as housekeeping genes, and the geometric means was computed for further calculation (*p* ≤ 0.05*; *p* ≤ 0.001**; *p* ≤ 0.001)

Gene	Geometric mean
*HK2*	85.42
*MITO*	162.57***
*COX-5B*	137.29^ T^
*GAPDH*	44.89**
*GLUT1*	113.64
*MCT1*	174.02*
*HIF-1α*	154.01^ T^
*VIM*	22.94***

### Nuclear and cytoplasmatic content of HIF-1α via immunofluorescence staining and western blot analysis

**Fig. 3:**
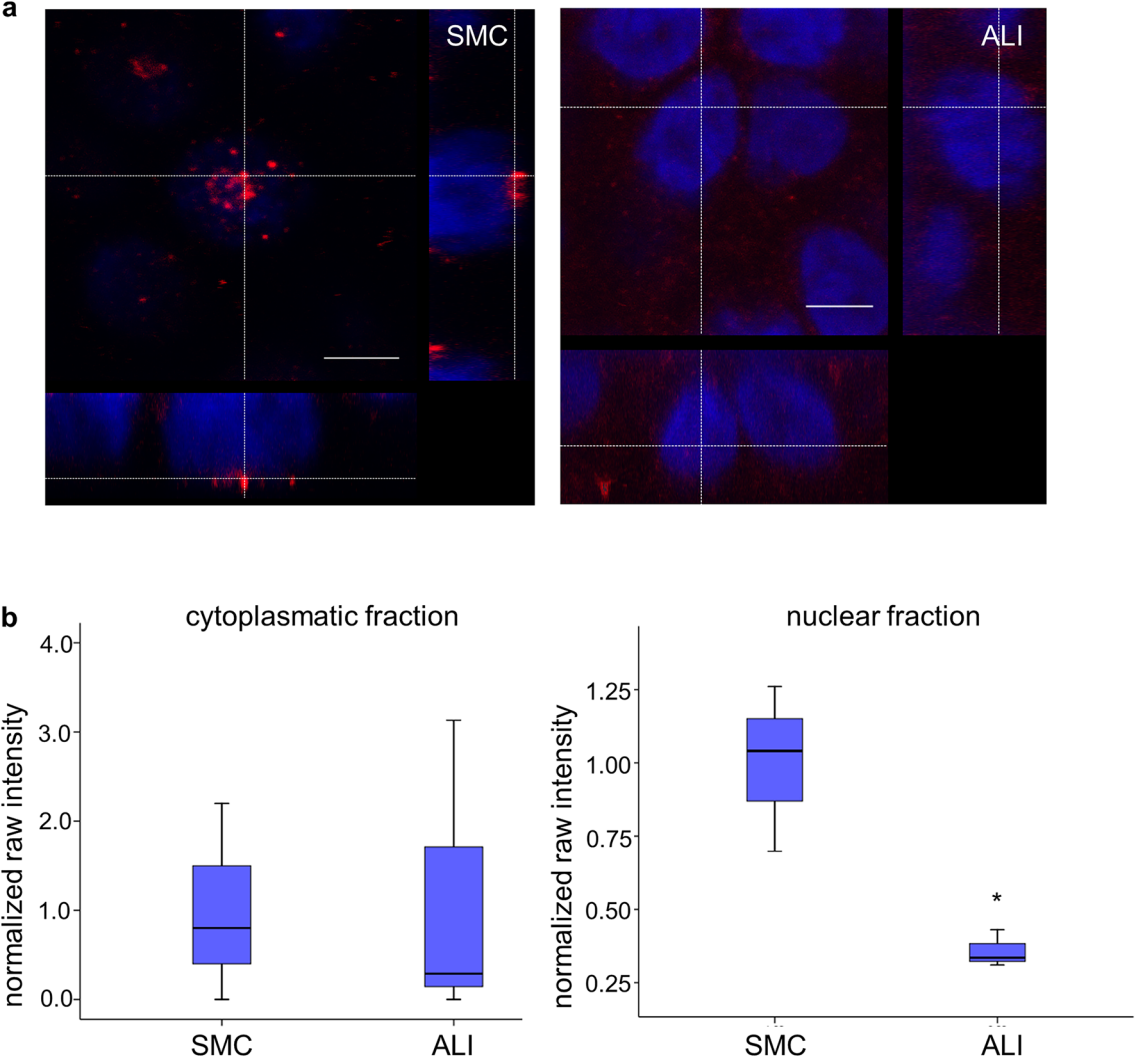
Analysis of HIF-1α protein. **a** HIF-1α was stained in SMC and ALI cultures of IPEC1 cells. We detected HIF-1α in close contact with the nucleus of SMC in contrast to ALI cultures. The cytoplasmatic fraction of ALI cultures showed a moderate staining for HIF-1α (blue, DAPI; red, HIF-1α; scale bar, 5 µm). **b** Proteins were separated with a kit in cytoplasmatic and nuclear fraction. No differences between the cytoplasmatic fraction of SMC- compared with ALI-cultured cells were found, but expression of HIF-1α was significantly decreased in the nuclear fraction of ALI compared with SMC (*p* < 0.05)

HIF-1α-mRNA (*p* = 0.093; Table 3) showed a trend of upregulation. On immunofluorescence staining, we detected HIF-1α in close contact to the nucleus in the SMC cultures but not in ALI cultures. In addition, we found a moderate staining of the cytoplasm in ALI (Fig. 3a). For western blot analyses, we used a kit for separation into nuclear and cytoplasmatic fractions. Subsequently, we used the loading control for normalization and statistically evaluated the normalized raw intensities. We detected no differences between the cytoplasmatic fraction of SMC and ALI, but a significantly lower HIF-1α content in the nuclear fraction of ALI cultures compared with SMC cultures (Fig. 3b). All original western blots of HIF-1α are in the supplementary material Fig. S2.

### Vimentin as marker for differentiation

**Fig. 4:**
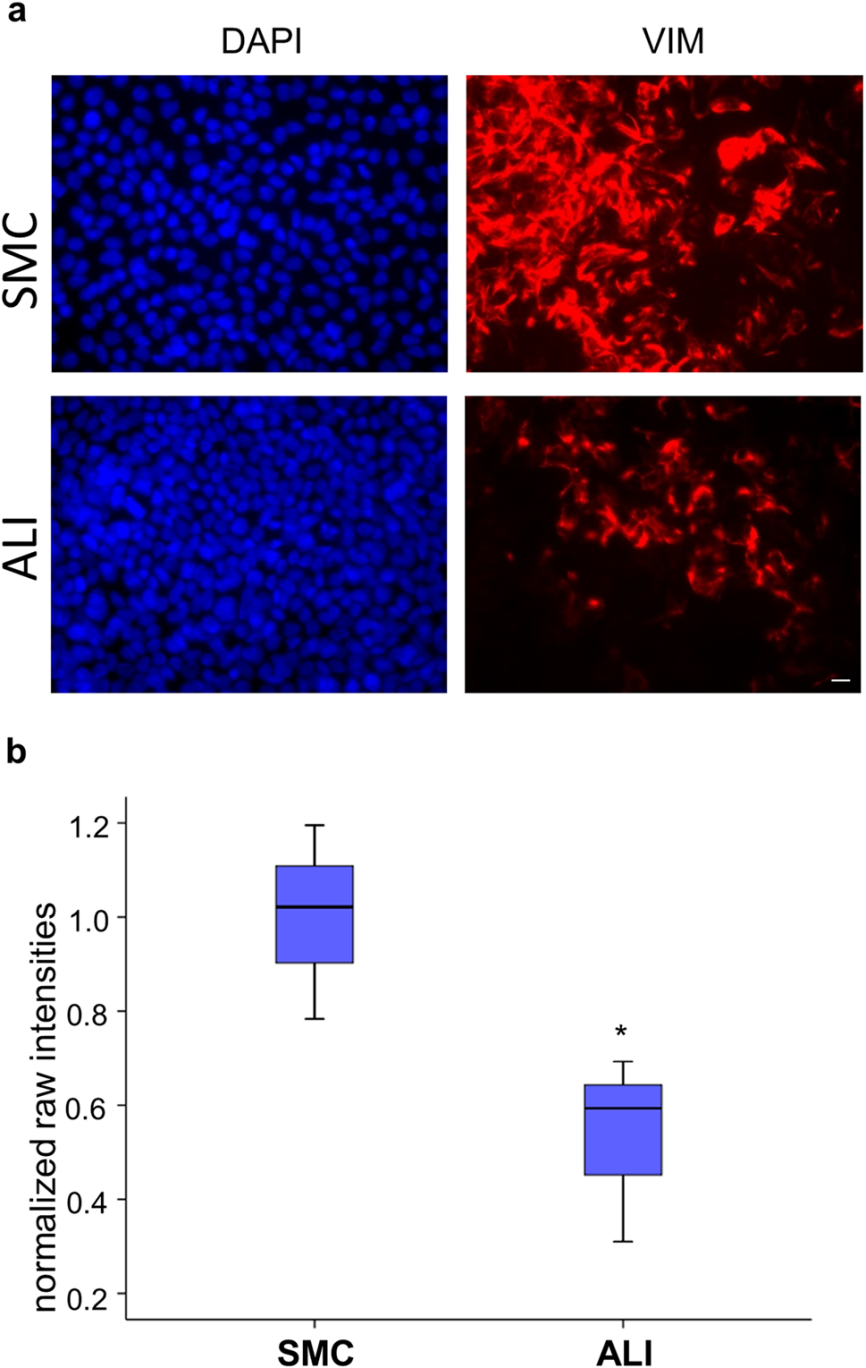
Analyses of vimentin. **a** SMC and ALI cultures were stained with mouse anti-vimentin (red) and DAPI (blue) for nucleus staining (bar, 10 µm). We detected a higher number of vimentin-positive cells in SMC than in ALI cultures. **b** SMC and ALI cultures were analyzed via western blot analyses (54 kDa). We used the raw intensities of the vimentin protein bands and the loading control β-actin for normalization for statistical evaluation. We observed a significant decreased vimentin protein expression (*p* < 0.05) in ALI compared with control (SMC)

Furthermore, we observed a downregulation of vimentin, as a marker for morphological differentiation, in ALI compared with SMC (22.94%; *p* ≤ 0.001; Table 3). In immunofluorescence staining (Fig. 4a) as well as in western blot analysis (Fig. 4b) we observed a higher vimentin content in SMC-cultured cells compared with ALI-cultured cells (*p* < 0.05). The original western blot can be found in the supplementary material as Fig. S3.

### Lactate and glucose measurements

**Fig. 5:**
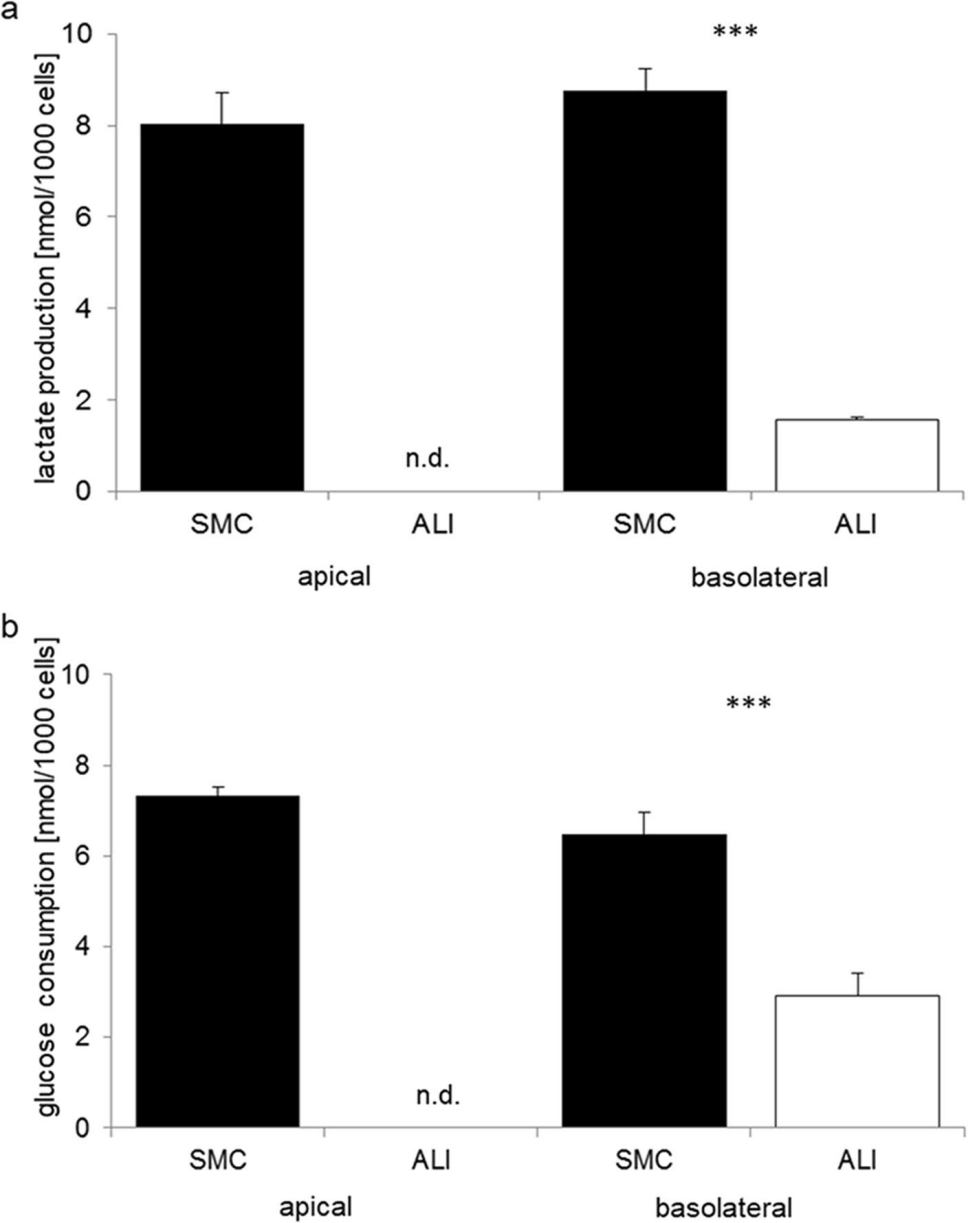
Lactate production (a) and glucose consumption (b). **a** Cells that were cultured as SMC showed significantly higher lactate production (9.17 nmol/1000 cells) compared with ALI (1.57 nmol/1000 cells) in the basolateral compartment (*p* < 0.001). **b** Furthermore, glucose consumption was measured, and a higher consumption was detected in SMC (6.47 nmol/1000 cells; *p* < 0.01). Lactate production and glucose consumption at the apical compartment of ALI culture was not determined (n.d.)

Furthermore, measurements of lactate production and glucose consumption are essential for the analysis of metabolic pathways. In our study, we observed a significantly higher lactate production in SMC (9.17 nmol lactate/1000 cells) compared with ALI (1.57 nmol/1000 cells; Fig. 5a) in the basolateral compartment (*p* ≤ 0.001). Similar results were found for glucose consumption. Here we observed in SMC 6.47 nmol/1000 cells and 2.92 nmol/1000 cells (*p* ≤ 0.001; Fig. 5b). ALI cultures used only 50% of the glucose compared with SMC cultures.

### Analysis of ATP content and cytochrome C oxidase activity

**Fig. 6:**
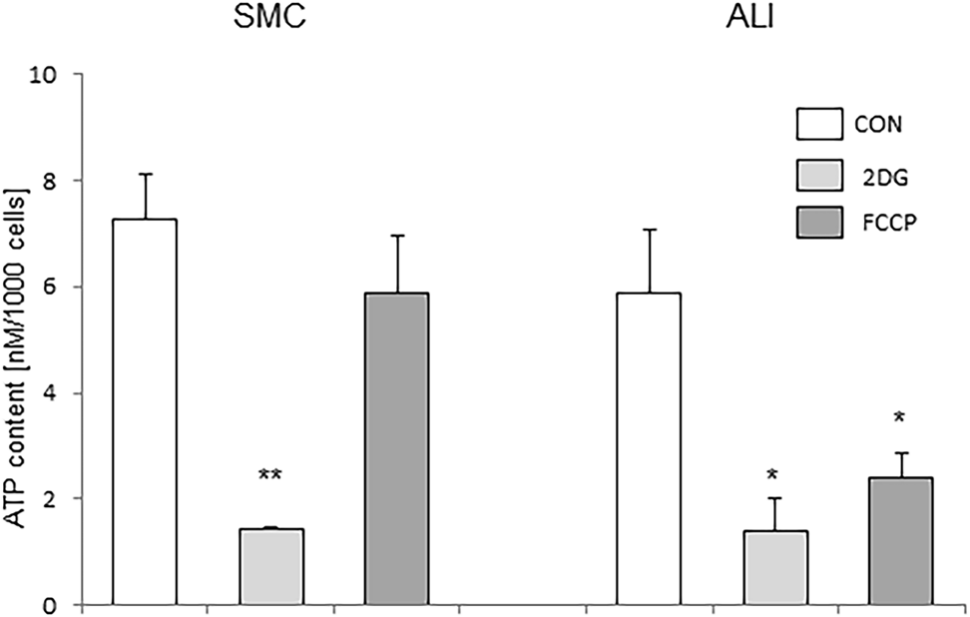
ATP analysis. No difference in ATP content of SMC (7.23 ± 0.87 nM/1000 cells) and ALI (5.86 ± 1.21 nM/1000 cells) cells was found but the treatment with different inhibitors of metabolism showed different results. A significant decreased ATP content was reached through blocking with 2DG in ALI (*p* = 0.021) and in SMC (*p* = 0.005). An uncoupling with FCCP resulted in a significant decrease of ATP in ALI (2.4 ± 0.46 nM/1000; *p* = 0.027) but not in SMC cells (5.86 ± 1.09 nM/1000; n.s.)

**Fig. 7:**
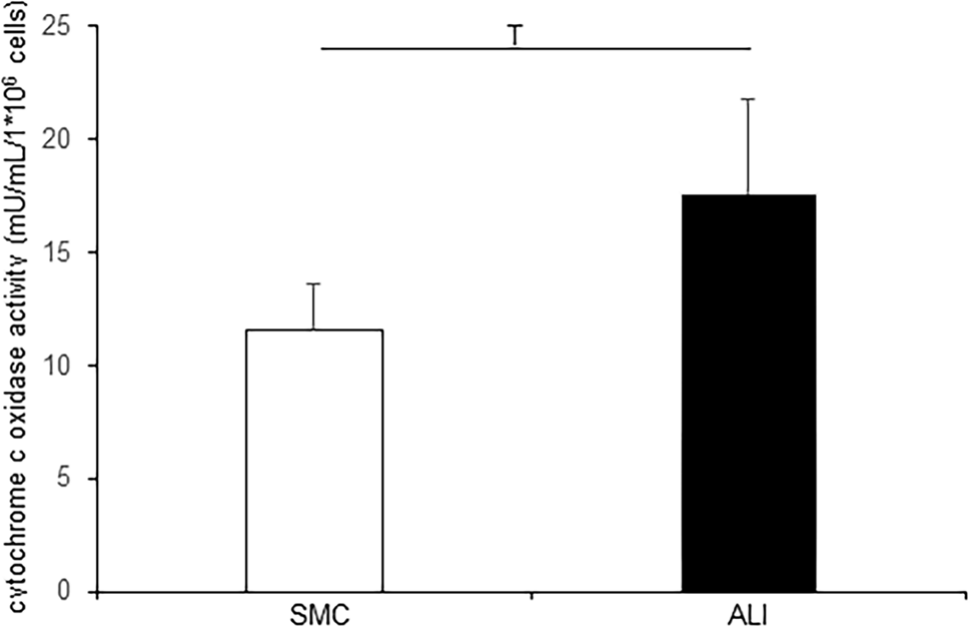
Cytochrome C oxidase activity. The cytochrome C oxidase activity was analyzed with a colorimetric assay based on the decrease of the absorbance at 550 nm. Here, cytochrome C oxidase of mitochondria oxidase ferrocytochrome C (reduced) to ferricytochrome C. ALI-cultured cells (17.63 ± 1.45 mU/mL/1 × 10^6^ cells; Fig. 6) showed a trend of a higher cytochrome c oxidase activity compared with SMC (11.55 ± 2.05 mU/mL/1*10^6^ cells; *p* = 0.069)

This resulted in an analysis of the ATP measurement and cytochrome C oxidase activity. Cells of submerged culture showed no difference in ATP content (7.23 ± 0.87 nM/1000 cells; Fig. 6) in comparison with ALI culture (5.86 ± 1.21 nM/1000 cells). An uncoupling with FCCP resulted in a significant decrease of ATP in ALI (2.4 ± 0.46 nM/1000 cells; *p* = 0.023) but not in SMC cells (5.86 ± 1.09 nM/1000 cells; n.s.). Furthermore, ALI culture (17.63 ± 1.45 mU/mL/1 × 10^6^ cells; Fig. 7) showed a trend of a higher cytochrome C oxidase activity compared with SMC (11.55 ± 2.05 mU/mL/1 × 10^6^ cells; *p* = 0.069).

## Discussion

An air–liquid interface culture improves oxygen consumption of intestinal epithelial cells (Klasvogt et al. 2017), and this is crucial since the gastrointestinal tract has one of the highest oxygen demands of the whole body, amounting to about 20–25% of the whole-body O_2_ consumption (Yen et al. 1989). The improved oxygen supply resulted in 31 significantly regulated KEGG pathways and *HIF* signaling ranked first among the pathways. This is not surprising, as *HIF* itself is oxygen regulated and is the main gene involved in the proceedings of the ALI cultures with a focus on metabolic shift (Klasvogt et al. 2017; Kondo et al. 1997; Xu et al. 2014). Under hypoxia, the HIF-1α subunit is stabilized and acts as a transcription factor in the nucleus. Here, it activates transcription of hypoxia-inducible genes, including those encoding hexokinase 1 and 2, aldolase A, enolase 1, lactate-dehydrogenase A, phosphofructokinase I, and glyceraldehyde-3-phosphate-dehydrogenase (Weidemann and Johnson 2008). These are important genes of glycolysis. This metabolic pathway is the second significantly altered pathway in our analyses. Glyceraldehyde-3-phosphatase is a key enzyme in glycolysis (2012) and is one of the most used housekeeping genes worldwide. Barber and colleagues probed 72 human tissues and found significant tissue-specific differences between them (Barber et al. 2005). In our study, *GAPDH* gene and protein was significantly downregulated in ALI compared with SMC cultures. Barber and colleagues inferred that a higher *GAPDH* expression is found in tissues with a higher energy requirement (Barber et al.^2005^).

In this study, we also observed significantly higher glucose consumption (SMC: 6.47 nmol/1000 cells; ALI: 2.92 nmol/1000 cells) and lactate production at the basolateral side of the SMC (9.17 nmol lactate/1000 cells) cells compared with ALI cells (1.57 nmol/1000 cells). This result supports the finding that there is increased glycolysis in the SMC compared with ALI. In vitro measurements of scraped mucosa showed high glucose metabolism and lactate production (Hanson and Parsons 1977; Watford et al. 1979). Vaugelade and colleagues (Vaugelade et al. 1994) found only slight differences between fed and fasted pigs. They isolated enterocytes of fasted and fed pigs and then added glucose and glutamine. They found an oxygen consumption of 1.12 nmol/min/1 × 10^6^ cells (fed pigs) and 1.23 nmol/min/1 × 10^6^ cells (fasted pigs). This was rounded through the measurement of lactate production: 0.24 nmol/min/1 × 10^6^ cells (fed pigs) and 0.46 nmol/min/1 × 10^6^ cells (fasted pigs). Our values differ from those of Vaugelade et al., but they used a suspension of isolated enterocytes and no adherent cells. Furthermore, we used a final point measurement for our study and no detection over time. The turnover from glucose to lactate was nearly at a ratio of 1:1 at the basolateral side of our cells.

The changes in metabolism could also be accompanied by differences in ATP content. In SMC and ALI we found similar ATP content (6–7 nM/1000 cells), but we could draw conclusions with the application of 2DG and FCCP on the pathways they use for energy production. 2DG resulted in a significant reduction of ATP content in both, but with FCCP no decreased content was observed in SMC compared with ALI. This also suggests that a higher glycolytic rate combined with a lower rate of oxidative phosphorylation occurred in SMC compared with ALI. This was, as already mentioned, confirmed by a higher GAPDH content in SMC. Nicholls and colleagues examined the role of GAPDH in apoptotic cells (Nicholls et al. 2012). A higher GAPDH content goes along with the initiation of apoptosis. In this study, we found a lower number of cells/µm^2^ in SMC. This could be due to a higher apoptotic rate in our cell culture system of SMC. This would indicate a pro-apoptotic effect of HIF-1α. However, other studies show an anti-apoptotic effect, which depends on cell line and conditions (Piret et al 2002). Consequently, an increased mitotic rate could play an important role in our ALI cultures. The analysis of the KEGG pathways also shows that there is an influence on the cell cycle (ninth significantly regulated pathway).

Furthermore, HIF-1α is a main target in cancer research (Talks et al. 2000; Zhong et al. 1999) because many genes that are induced by HIF-1α are expressed at higher levels in cancer than in normal tissues such as enzymes of glucose metabolism. This goes along with an increased glucose uptake and lactate production, and decreased respiration. The SMC cultures of IPEC-1 appear to suffer from insufficient oxygen supply under standard conditions. We can possibly simulate the first step in cancer development because they show an increased glucose uptake, increased lactate production, increased nuclear HIF-1α, and decreased O_2_ consumption (data not shown) compared with ALI cultures. Consequently, ALI cultures tend to reflect the characteristics of healthy and optimally supplied enterocytes. Different cancer therapeutics such as gemcitabine showed a higher antitumor effect in combination with an inhibitor of HIF-1α “PX-478” than alone (Zhao et al. 2015). Furthermore, radiation is one of the standard treatments for patients with locally advanced rectal cancer and allows tumor reduction and decreases local recurrence (Pucciarelli et al. 2004; Sauer et al. 2004). Okuno and colleagues examined the effect of radiation and SN-38 (Okuno et al. 2018) on HIF-1α. Radiation itself causes a dose-dependent upregulation of HIF-1α (Moeller et al. 2004; Okuno et al. 2018), and SN-38 blocks the upregulation caused by radiation (Okuno et al. 2018). A slight criticism of their study is their data on of HIF-1α because they did not separate their proteins into nuclear and cytoplasmatic fractions. Furthermore, IPEC-1 and IPEC-J2 seem to be differently regulated on the mRNA level because IPEC-J2 showed a downregulation of HIF-1α-mRNA in ALI combined with a decrease of nuclear protein content (Klasvogt et al. 2017). On the other hand, in IPEC-1 we observed a trend of upregulation of *HIF-1α* mRNA. However, both cell lines show significantly lower HIF-1 content in the nucleus of ALI-cultured cells compared with SMC-cultured cells.

## Conclusion

Further analyses should be done with a focus on subunits and isoforms of the HIF complex, but we suggest that this is a good starting point for pharmacological manipulation of HIF-1α in the small intestine with two different culture systems: (1) SMC activates HIF-1α and (2) ALI inhibits HIF-1α. In this context, this study may offer new starting points for further research into the development of cancer in the small intestine. Here, the volcano plot analysis sheds light on some genes, including *RGS5*, *BBOX1*, *PKHD1L1*, and *TXRND3*, that are associated with cancer.

## Supplementary Information

Below is the link to the electronic supplementary material.

Supplementary file1 (PDF 255 KB)

Supplementary file2 (PDF 216 KB)

Supplementary file3 (PDF 165 KB)

Supplementary file4 (PDF 73 KB)

## Abbreviations

ALI: Air–liquid interface culture
BBOX1: γ-Butyrobetaine hydroxylase
CO_2_: Carbon dioxide
COL3A1: Collagen type III alpha 1 chain
COL5A2: Collagen type V alpha 2 chain
COX-5B: Cytochrome C oxidase subunit 5B
CNTN6: Contacting 6
2DG: 2-Desoxy-d-glucose
FAO: Fatty acid oxidation
FBS: Fetal bovine serum
FCCP: 2-[2-[4-(Trifluoromethoxy)phenyl]hydrazinylidene]-propanedinitrile
GLUT1: Glucose transporter 1
HIF: Hypoxia-inducible factor
HK2: Hexokinase
IPEC-1: Intestinal porcine epithelial cells 1 (origin: jejunum and ileum)
IPEC-J2: Intestinal porcine epithelial cells (origin: jejunum)
ITS: Insulin–transferrin–sodium selenite
KCl: Potassium chloride
MCT1: Monocarboxylate transporter 1
PB: Phosphate buffer
PKHD1L1: Polycystic Kidney and Hepatic Disease 1 (autosomal recessive)-Like 1
PVDF: Polyvinylidenfluoride
qPCR: Quantitative PCR
RGS5: Regulator of G protein 5
RT: Room temperature
SMC: Submerged culture
T: Trend
TEER: Transepithelial electrical resistance
TFF3: Trefoil factor 3
TNBC: Triple-negative breast cancer
TXNRD3: Thioredoxin reductase 3
VIM: Vimentin

## References

[CR1] Barber RD, Harmer DW, Coleman RA, Clark BJ (2005). GAPDH as a housekeeping gene: analysis of GAPDH mRNA expression in a panel of 72 human tissues. Physiol Genomics.

[CR2] Brandes RP, Rezende F (2021). Glycolysis and inflammation partners in crime. Circ Res.

[CR3] Cozens, D., E. Sutherland, F. Marchesi, G. Taylor, C.C. Berry, R.L. Davies. (2018) Temporal differentiation of bovine airway epithelial cells grown at an air-liquid interface. Sci Rep-Uk 8 10.1038/s41598-018-33180-w PMC6173764 30291311

[CR4] Diesing AK, Nossol C, Ponsuksili S, Wimmers K, Kluess J, Walk N, Post A, Rothkotter HJ, Kahlert S (2012). Gene regulation of intestinal porcine epithelial cells IPEC-J2 is dependent on the site of deoxynivalenol toxicological action. PLoS ONE.

[CR5] Edgar R, Domrachev M, Lash AE (2002). Gene expression omnibus: NCBI gene expression and hybridization array data repository. Nucleic Acids Res.

[CR6] Geens MM, Niewold TA (2011). Optimizing culture conditions of a porcine epithelial cell line IPEC-J2 through a histological and physiological characterization. Cytotechnology.

[CR8] Guilloteau P, Zabielski R, Hammon HM, Metges CC (2010). Nutritional programming of gastrointestinal tract development. Is the pig a good model for man?. Nutr Res Rev.

[CR9] Hanson PJ, Parsons S (1977). Metabolism and transport of glutamine and glucose in vascularly perfused small intestine rat. Biochem J.

[CR10] Kauffmann A, Gentleman R, Huber W (2009). arrayquality metrics—a bioconductor package for quality assessment of microarray data. Bioinformatics.

[CR11] Klasvogt, S., W. Zuschratter, A. Schmidt, A. Krober, S. Vorwerk, R. Wolter, B. Isermann, K. Wimmers, H.J. Rothkotter, and C. Nossol. (2017) Air-liquid interface enhances oxidative phosphorylation in intestinal epithelial cell line IPEC-J2. Cell Death Discov 3 10.1038/cddiscovery.2017.1 PMC5327501 28250970

[CR12] Kondo M, Tamaoki J, Sakai A, Kameyama S, Kanoh S, Konno K (1997). Increased oxidative metabolism in cow tracheal epithelial cells cultured at air-liquid interface. Am J Resp Cell Mol.

[CR13] Moeller BJ, Cao YT, Li CY, Dewhirst MW (2004). Radiation activates HIF-1 to regulate vascular radiosensitivity in tumors: Role of reoxygenation, free radicals, and stress granules. Cancer Cell.

[CR14] Nicholls, C., H. Li, J.P. Liu. 2012. GAPDH: A common enzyme with uncommon functions. *Clin Exp Pharmacol P*. 39:674–679. 10.1111/j.1440-1681.2011.05599.x 21895736

[CR15] Nossol C, Diesing AK, Walk N, Faber-Zuschratter H, Hartig R, Post A, Kluess J, Rothkotter HJ, Kahlert S (2011). Air-liquid interface cultures enhance the oxygen supply and trigger the structural and functional differentiation of intestinal porcine epithelial cells (IPEC). Histochem Cell Biol.

[CR16] Okuno T, Kawai K, Hata K, Murono K, Emoto S, Kaneko M, Sasaki K, Nishikawa T, Tanaka T, Nozawa H (2018). SN-38 acts as a radiosensitizer for colorectal cancer by inhibiting the radiation-induced up-regulation of HIF-1 alpha. Anticancer Res.

[CR17] Ootani A, Toda S, Fujimoto K, Sugihara H (2000). An air-liquid interface promotes the differentiation of gastric surface mucous cells (GSM06) in culture. Biochem Bioph Res Co.

[CR18] Piret J-P, Mottet D, Raes M, Michiels C (2002). Is HIF-1α a pro- or an anti-apoptotic protein?. Biochem Pharm.

[CR19] Pucciarelli S, Toppan P, Friso ML, Russo V, Pasetto L, Urso E, Marino F, Ambrosi A, Lise M (2004). Complete pathologic response following preoperative chemoradiation therapy for middle to lower rectal cancer is not a prognostic factor for a better outcome. Dis Colon Rectum.

[CR20] Sauer R, Becker H, Hohenberger W, Rodel C, Wittekind C, Fietkau R, Martus P, Tschmelitsch J, Hager E, Hess CF, Karstens JH, Liersch T, Schmidberger H, Raab R, Grp GRCS (2004). Preoperative versus postoperative chemoradiotherapy for rectal cancer. New Engl J Med.

[CR22] Steube KG, Koelz AL, Uphoff CC, Drexler HG, Kluess J, Steinberg P (2012). The necessity of identity assessment of animal intestinal cell lines: a case report. Cytotechnology.

[CR23] Talks KL, Turley H, Gatter KC, Maxwell PH, Pugh CW, Ratcliffe PJ, Harris AL (2000). The expression and distribution of the hypoxia-inducible factors HIF-1 alpha and HIF-2 alpha in normal human tissues, cancers, and tumor-associated macrophages. Am J Pathol.

[CR24] Vaugelade P, Posho L, Darcyvrillon B, Bernard F, Morel MT, Duee PH (1994). Intestinal oxygen uptake and glucose metabolism during nutrient absorption in the pig. Proc Soc Exp Biol Med.

[CR25] Watford M, Lund P, Krebs HA (1979). Isolation and metabolic characteristics of rat and chicken enterocytes. Biochem J.

[CR26] Weidemann A, Johnson RS (2008). Biology of HIF-1 alpha. Cell Death Differ.

[CR27] Xu WL, Janocha AJ, Leahy RA, Klatte R, Dudzinski D, Mavrakis LA, Comhair SAA, Lauer ME, Cotton CU, Erzurum SC (2014). A novel method for pulmonary research: assessment of bioenergetic function at the air-liquid interface. Redox Biol.

[CR28] Yen, J.T., J.A. Nienaber, D.A. Hill, and W.G. Pond. 1989. Oxygen consumption by portal vein-drained organs and by whole animal in conscious growing swine. Proceedings of the Society for Experimental Biology and Medicine. Society for Experimental Biology and Medicine. 190 393–398 10.3181/00379727-190-42878 2928354

[CR29] Zabielski R, Godlewski MM, Guilloteau P (2008). Control of development of gastrointestinal system in neonates. J Physiol Pharmacol.

[CR30] Zhao TS, Ren H, Jia L, Chen J, Xin W, Yan F, Li J, Wang XC, Gao S, Qian D, Huang CB, Hao JH (2015). Inhibition of HIF-1 alpha by PX-478 enhances the anti-tumor effect of gemcitabine by inducing immunogenic cell death in pancreatic ductal adenocarcinoma. Oncotarget.

[CR31] Zhong H, De Marzo AM, Laughner E, Lim M, Hilton DA, Zagzag D, Buechler P, Isaacs WB, Semenza GL, Simons JW (1999). Overexpression of hypoxia-inducible factor 1 alpha in common human cancers and their metastases. Cancer Res.

