# Supplementary material for: Air–liquid interface cultures trigger a metabolic shift in intestinal epithelial cells (IPEC-1)
Source: Histochem Cell Biol. 2023 Feb 15;159(5):389–400. doi: 10.1007/s00418-023-02180-x (PMC10191962; doi:10.1007/s00418-023-02180-x)
Supplement: Supplementary file 1 — Supplementary file1 (PDF 255 KB) [file 418_2023_2180_MOESM1_ESM.pdf]

Air-liquid interface cultures trigger a metabolic shift in intestinal epithelial cells (IPEC-1)

Martin Stollmeier[1], Stefan Kahlert[1], Werner Zuschratter[2], Michael Oster[3], Klaus Wimmers[3], Berend Isermann[4], Hermann-Josef Rothkötter[1], Constanze Nossol[1]

corresponding author:
Dr. rer. nat. Constanze Nossol
Institute of Anatomy Medical Faculty
Otto-von-Guericke University
39120 Magdeburg
Germany

Fig. S1 Analyses of different genes via western blot. This figure is related to **Fig. 2.** Different genes COX5B [14 kDa] (**a**), MCT1 [54 kDa] (**b**), MITO [60 kDa] (**c**) and GAPDH [37 kDa] (**d**) were analysed via western blot analysis. 3 independent experiments (passage 116, 117, 118) were carried out. **aa, bb, cc, dd** In addition, β-actin was used as loading control (42 kDa).

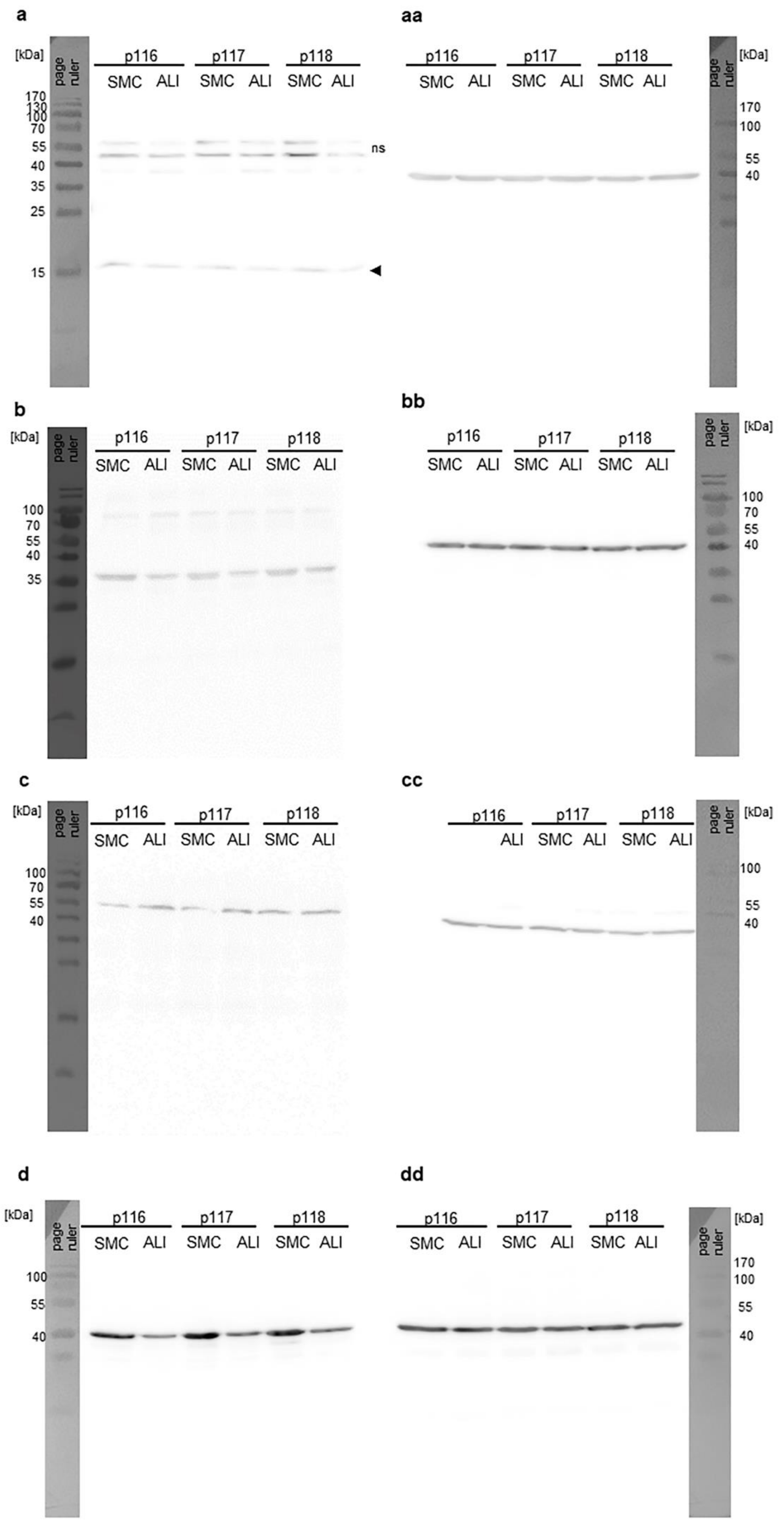
a
aa
b
bb
c
cc
d
dd
[kDa]
page ruler
p116
p117
p118
SMC
ALI
ns
170
130
100
70
55
40
35
25
15
