# Supplementary material for: Air–liquid interface cultures trigger a metabolic shift in intestinal epithelial cells (IPEC-1)
Source: Histochem Cell Biol. 2023 Feb 15;159(5):389–400. doi: 10.1007/s00418-023-02180-x (PMC10191962; doi:10.1007/s00418-023-02180-x)
Supplement: Supplementary file 2 — Supplementary file2 (PDF 216 KB) [file 418_2023_2180_MOESM2_ESM.pdf]

Air-liquid interface cultures trigger a metabolic shift in intestinal epithelial cells (IPEC-1)

Martin Stollmeier[1], Stefan Kahlert[1], Werner Zuschratter[2], Michael Oster[3], Klaus Wimmers[3], Berend Isermann[4], Hermann-Josef Rothkötter[1], Constanze Nossol[1]

corresponding author:
Dr. rer. nat. Constanze Nossol
Institute of Anatomy Medical Faculty
Otto-von-Guericke University
39120 Magdeburg
Germany

Fig. S2 Proteins were separated in cytoplasmatic and nuclear fraction with the NE-PER Nuclear and Cytoplasmic Extraction Reagents. This figure is related to **Fig. 3**. We demonstrate the original western blots of all 4 experiments (passage 113, 114, 115 and 119). **a, b, c** A higher content of HIF-1α (predicted size: 93 kDa) was detected in the nuclear cytoplasmatic fraction of SMC compared to ALI. Hela cells were used as control cells and were treated with 100 uM $CoCl_2$ for 4 hours to induce hypoxia. The cell lysate was used as control for HIF-1α expression but was purchased from Nobus Biologicals (USA). **aa, bb, cc** β-actin was used as loading control (42 kDa). In b and bb a loading of the nuclear fraction (*; p114) was not possible, because protein content of the sample was too low.

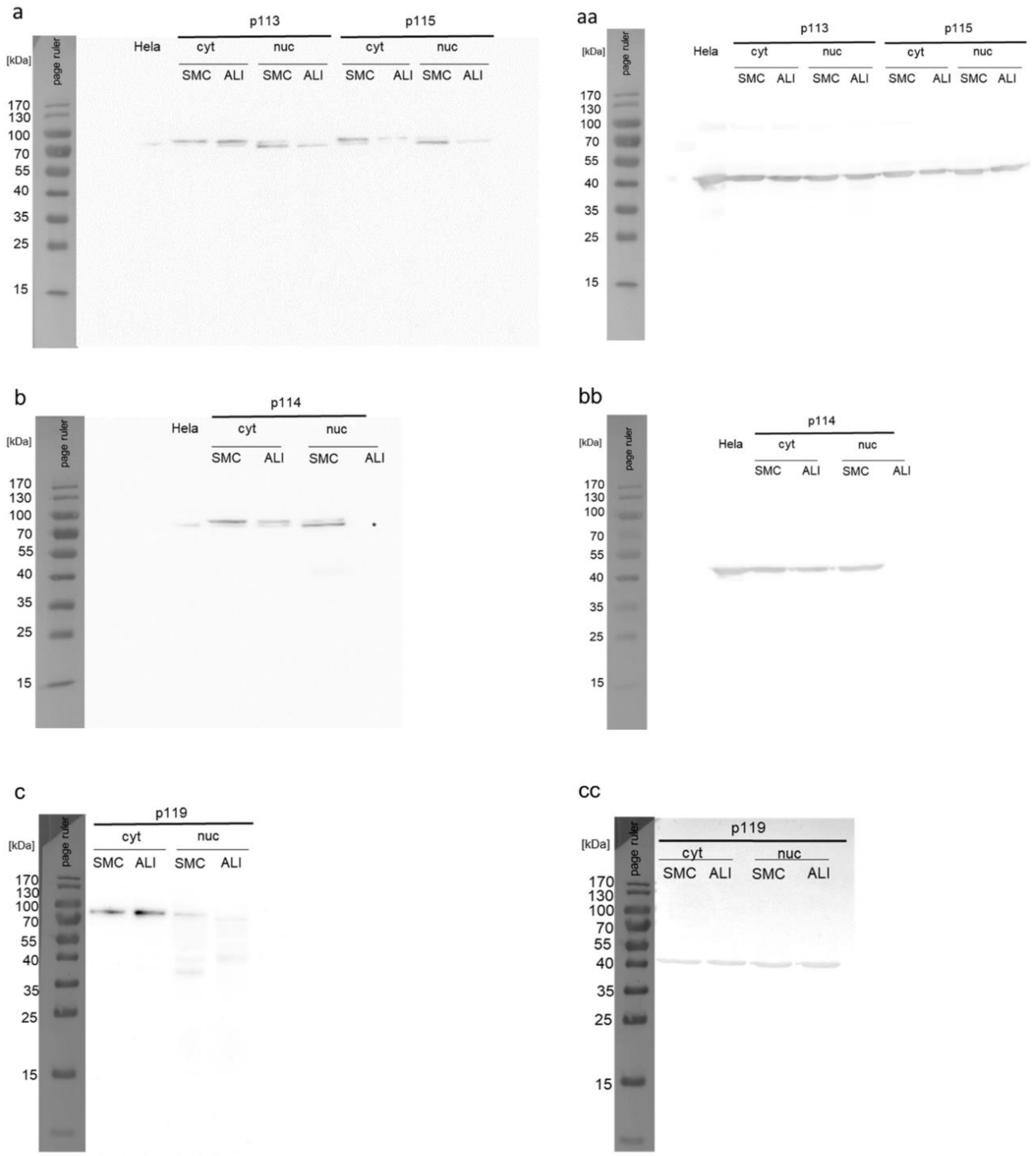
a
p113
p115
Hela
cyt
nuc
SMC
ALI
[kDa]
page ruler
170
130
100
70
55
40
35
25
15
aa
b
p114
bb
c
p119
cc
