# Supplementary figures and images for: Air–liquid interface cultures trigger a metabolic shift in intestinal epithelial cells (IPEC-1)
Source: Histochem Cell Biol. 2023 Feb 15;159(5):389–400. doi: 10.1007/s00418-023-02180-x (PMC10191962; doi:10.1007/s00418-023-02180-x)

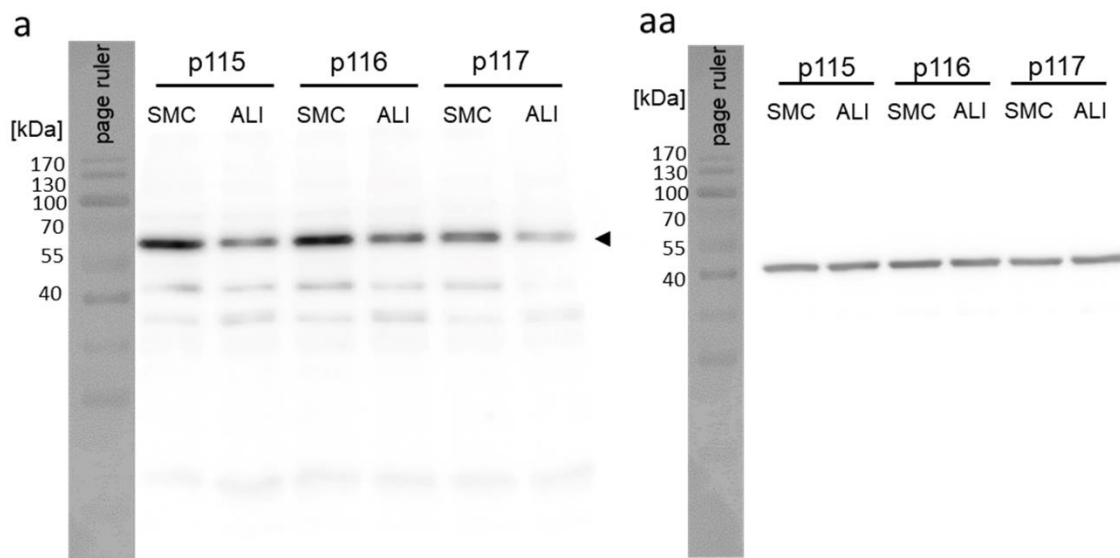

Supplement: Supplementary file 3 — Supplementary file3 (PDF 165 KB) [file 418_2023_2180_MOESM3_ESM.pdf]
