# Supplementary material for: Air–liquid interface cultures trigger a metabolic shift in intestinal epithelial cells (IPEC-1)
Source: Histochem Cell Biol. 2023 Feb 15;159(5):389–400. doi: 10.1007/s00418-023-02180-x (PMC10191962; doi:10.1007/s00418-023-02180-x)
Supplement: Supplementary file 4 — Supplementary file4 (PDF 73 KB) [file 418_2023_2180_MOESM4_ESM.pdf]

Air-liquid interface cultures trigger a metabolic shift in intestinal epithelial cells (IPEC-1)

Martin Stollmeier[1], Stefan Kahlert[1], Werner Zuschratter[2], Michael Oster[3], Klaus Wimmers[3], Berend Isermann[4], Hermann-Josef Rothkötter[1], Constanze Nossol[1]

corresponding author:
Dr. Constanze Nossol
Institute of Anatomy Medical Faculty
Otto-von-Guericke University
39120 Magdeburg
Germany

Tab. S1 The table contains a list of the significantly regulated genes of the top 10 regulated KEGG-pathways.

gene	HIF-signaling pathway
1	MAPK interacting serine/threonine kinase 2(MKNK2)
2	aldolase, fructose-bisphosphate C(ALDOC)
3	cyclin dependent kinase inhibitor 1A(CDKN1A)
4	egl-9 family hypoxia inducible factor 1(EGLN1)
5	egl-9 family hypoxia inducible factor 3(EGLN3)
6	enolase 1(ENO1)
7	eukaryotic translation initiation factor 4E binding protein 1(EIF4EBP1)
8	fms related receptor tyrosine kinase 1(FLT1)
9	glyceraldehyde-3-phosphate dehydrogenase(GAPDH)
10	hexokinase 1(HK1)
11	inhibitor of carbonic anhydrase(INHCA)
12	interleukin 6 receptor(IL6R)
13	lactate dehydrogenase A(LDHA)
14	phosphofructokinase, liver type(PFKL)
15	phosphoglycerate kinase 1(PGK1)
16	toll like receptor 4(TLR4)
17	transferrin(TF)

gene	glycolysis/gluconeogenesis
1	acyl-CoA synthetase short chain family member 2(ACSS2)
2	aldehyde dehydrogenase 2 family member(ALDH2)
3	aldolase, fructose-bisphosphate C(ALDOC)

4	enolase 1(ENO1)
5	glucose-6-phosphate isomerase(GPI)
6	glyceraldehyde-3-phosphate dehydrogenase(GAPDH)
7	hexokinase 1(HK1)
8	lactate dehydrogenase A(LDHA)
9	phosphofructokinase, liver type(PFKL)
10	phosphoglucomutase 1(PGM1)
11	phosphoglycerate kinase 1(PGK1)
12	pyruvate kinase, muscle(PKM)
13	triosephosphate isomerase 1(TPI1)

gene	metabolic pathways
1	3'-phosphoadenosine 5'-phosphosulfate synthase 1(PAPSS1)
2	3'-phosphoadenosine 5'-phosphosulfate synthase 2(PAPSS2)
3	3-hydroxy-3-methylglutaryl-CoA synthase 1(HMGCS1)
4	7-dehydrocholesterol reductase(DHCR7)
5	ATPase H+ transporting V1 subunit A(ATP6V1A)
6	ATPase H+/K+ transporting non-gastric alpha2 subunit(ATP12A)
7	ChaC glutathione specific gamma-glutamylcyclotransferase 1(CHAC1)
8	EBP cholestenol delta-isomerase(EBP)
9	L-threonine dehydrogenase(TDH)
10	NAD(P)H quinone dehydrogenase 1(NQO1)
11	ST3 beta-galactoside alpha-2,3-sialyltransferase 1(ST3GAL1)
12	UDP-GlcNAc:betaGal beta-1,3-N-acetylglucosaminyltransferase 2(B3GNT2)
13	acyl-CoA synthetase long chain family member 1(ACSL1)
14	acyl-CoA synthetase short chain family member 2(ACSS2)
15	adenosylhomocysteinase like 1(AHCYL1)
16	adenylosuccinate synthase 1(ADSS1)
17	aldehyde dehydrogenase 2 family member(ALDH2)
18	aldolase, fructose-bisphosphate C(ALDOC)
19	alkaline ceramidase 3(ACER3)
20	beta-1,3-N-acetylgalactosaminyltransferase 1(B3GALNT1)
21	beta-1,4-galactosyltransferase 6(B4GALT6)
22	branched chain amino acid transaminase 1(BCAT1)
23	carbonyl reductase 1(CBR1)
24	carbonyl reductase 2(CBR2)
25	catalase(CAT)
26	creatine kinase B(CKB)
27	cytidine deaminase(CDA)
28	cytochrome P450 family 2 subfamily J member 93(CYP2J93)
29	dihydropyrimidine dehydrogenase(DPYD)
30	dopa decarboxylase(DDC)
31	ectonucleoside triphosphate diphosphohydrolase 4(ENTPD4)
32	enolase 1(ENO1)
33	farnesyl-diphosphate farnesyltransferase 1(FDFT1)
34	fatty acid desaturase 1(FADS1)

35 flavin containing dimethylaniline monoxygenase 1(FMO1)
36 galactosidase alpha(GLA)
37 glucosaminyl (N-acetyl) transferase 4(GCNT4)
38 glucose-6-phosphate isomerase(GPI)
39 glutaminase(GLS)
40 glutathione S-transferase A4(LOC100510917)
41 glutathione S-transferase alpha 4(GSTA4)
42 glutathione S-transferase omega 1(GSTO1)
43 glyceraldehyde-3-phosphate dehydrogenase(GAPDH)
44 hexokinase 1(HK1)
45 hypoxanthine phosphoribosyltransferase 1(HPRT1)
46 lactate dehydrogenase A(LDHA)
47 lipin 2(LPIN2)
48 malic enzyme 1(ME1)
49 mannose phosphate isomerase(MPI)
50 mannosidase alpha class 1C member 1(MAN1C1)
51 membrane bound O-acyltransferase domain containing 1(MBOAT1)
52 methylsterol monooxygenase 1(MSMO1)
53 myotubularin related protein 2(MTMR2)
54 neuraminidase 1(NEU1)
55 nicotinamide phosphoribosyltransferase(NAMPT)
56 ornithine aminotransferase(OAT)
57 pancreatic lipase related protein 2(PNLIPRP2)
58 phosphodiesterase 7A(PDE7A)
59 phosphofructokinase, liver type(PFKL)
60 phosphoglucomutase 1(PGM1)
61 phosphoglucomutase 3(PGM3)
62 phosphoglycerate kinase 1(PGK1)
63 phospholipase A and acyltransferase 3(PLAAT3)
64 phosphoserine phosphatase(PSPH)
65 polypeptide N-acetylgalactosaminyltransferase 1(GALNT1)
66 procollagen-lysine,2-oxoglutarate 5-dioxygenase 1(PLOD1)
67 procollagen-lysine,2-oxoglutarate 5-dioxygenase 2(PLOD2)
68 prolyl 4-hydroxylase subunit alpha 1(P4HA1)
69 prolyl 4-hydroxylase subunit alpha 2(P4HA2)
70 pyruvate carboxylase(PC)
71 pyruvate kinase, muscle(PKM)
72 selenophosphate synthetase 2(SEPHS2)
73 spermine oxidase(SMOX)
74 stearoyl-CoA desaturase(SCD)
75 sterol-C5-desaturase(SC5D)
76 sulfotransferase family 1E, estrogen-preferring, member 1(SULT1E1)
77 thiosulfate sulfurtransferase(TST)
78 triosephosphate isomerase 1(TPI1)
79 uridine phosphorylase 1(UPP1)
80 uroporphyrinogen decarboxylase(UROD)

gene	carbon metabolism
1	acyl-CoA synthetase short chain family member 2(ACSS2)
2	aldolase, fructose-bisphosphate C(ALDOC)
3	catalase(CAT)
4	enolase 1(ENO1)
5	glucose-6-phosphate isomerase(GPI)
6	glyceraldehyde-3-phosphate dehydrogenase(GAPDH)
7	hexokinase 1(HK1)
8	malic enzyme 1(ME1)
9	phosphofructokinase, liver type(PFKL)
10	phosphoglycerate kinase 1(PGK1)
11	phosphoserine phosphatase(PSPH)
12	pyruvate carboxylase(PC)
13	pyruvate kinase, muscle(PKM)
14	triosephosphate isomerase 1(TPI1)

gene	biosynthesis of amino acids
1	aldolase, fructose-bisphosphate C(ALDOC)
2	branched chain amino acid transaminase 1(BCAT1)
3	enolase 1(ENO1)
4	glyceraldehyde-3-phosphate dehydrogenase(GAPDH)
5	phosphofructokinase, liver type(PFKL)
6	phosphoglycerate kinase 1(PGK1)
7	phosphoserine phosphatase(PSPH)
8	pyruvate carboxylase(PC)
9	pyruvate kinase, muscle(PKM)
10	triosephosphate isomerase 1(TPI1)

gene	biosynthesis of amino acids
1	7-dehydrocholesterol reductase(DHCR7)
2	EBP cholestenol delta-isomerase(EBP)
3	farnesyl-diphosphate farnesyltransferase 1(FDFT1)
4	methylsterol monooxygenase 1(MSMO1)
5	sterol-C5-desaturase(SC5D)

gene	cholesterol metabolism
1	ATP binding cassette subfamily A member 1(ABCA1)
2	NPC intracellular cholesterol transporter 2(NPC2)
3	apolipoprotein H(APOH)
4	cell death inducing DFFA like effector b(CIDEB)
5	low density lipoprotein receptor(LDLR)
6	myosin regulatory light chain interacting protein(MYLIP)
7	voltage dependent anion channel 3(VDAC3)

gene	pyruvate metabolism
1	acyl-CoA synthetase short chain family member 2(ACSS2)

**2**	aldehyde dehydrogenase 2 family member(ALDH2)
**3**	lactate dehydrogenase A(LDHA)
**4**	malic enzyme 1(ME1)
**5**	pyruvate carboxylase(PC)
**6**	pyruvate kinase, muscle(PKM)

gene	cell cycle
**1**	PTTG1 regulator of sister chromatid separation, securin(PTTG1)
**2**	SMAD family member 2(SMAD2)
**3**	WEE1 G2 checkpoint kinase(WEE1)
**4**	cell division cycle 20(CDC20)
**5**	cyclin A2(CCNA2)
**6**	cyclin B1(CCNB1)
**7**	cyclin B2(CCNB2)
**8**	cyclin dependent kinase inhibitor 1A(CDKN1A)
**9**	cyclin dependent kinase inhibitor 2D(CDKN2D)
**10**	extra spindle pole bodies like 1, separase(ESPL1)

gene	drug-metabolism - other enzymes
**1**	cytidine deaminase(CDA)
**2**	dihydropyrimidine dehydrogenase(DPYD)
**3**	glutathione S-transferase A4(LOC100510917)
**4**	glutathione S-transferase alpha 4(GSTA4)
**5**	glutathione S-transferase omega 1(GSTO1)
**6**	hypoxanthine phosphoribosyltransferase 1(HPRT1)
**7**	uridine phosphorylase 1(UPP1)
